# Neurobiology and clinical features of impulse control failure in Parkinson’s disease

**DOI:** 10.1186/s42466-019-0013-5

**Published:** 2019-03-20

**Authors:** Matthieu Béreau, Paul Krack, Norbert Brüggemann, Thomas F. Münte

**Affiliations:** 1 Department of Neurology, University Hospital of Besançon, 25030 Besançon, Cedex France; 2 Department of Neurology, Inselspital, University of Bern, CH-3010 Bern, Switzerland; 3 Department of Neurology, University of Lübeck, 23562 Lübeck, Germany; 4 Institute of Psychology II, University of Lübeck, 23562 Lübeck, Germany

**Keywords:** Parkinson’s disease, Impulse control disorders, Behavioral addictions, Dopamine, Neuropsychiatric fluctuations, Mesocorticolimbic sensitization

## Abstract

Impulse control disorders (ICDs) and other impulsive-compulsive related behaviours are frequent and still under recognized non-motor complications of Parkinson’s disease (PD). They result from sensitization of the mesocorticolimbic pathway that arose in predisposed PD patients concomitantly with spreading of PD pathology, non-physiological dopaminergic and pulsatile administration of dopamine replacement therapy (DRT). Neuropsychiatric fluctuations (NPF) reflect the psychotropic effects of dopaminergic drugs and play a crucial role in the emergence of ICDs and behavioral addictions. Dopamine agonists (DA) which selectively target D2 and D3 receptors mostly expressed within the mesocorticolimbic pathway, are the main risk factor to develop ICDs. Neuroimaging studies suggest that dopamine agonists lead to a blunted response of the brain’s reward system both during reward delivery and anticipation. Genetic predispositions are crucial for the responsiveness of the mesolimbic system and the development of ICDs with several genes having been identified. Early screening for neuropsychiatric fluctuations, reduction of DA, fractionating levodopa dosage, education of patients and their relatives, are the key strategies for diagnosis and management of ICDs and related disorders.

## Background

Although motor signs namely bradykinesia, rigidity and tremor are still considered as the core feature of diagnostic criteria for Parkinson disease (PD) [71], increasing recognition has been given over time to non-motor manifestations of PD including cognitive, autonomic, and neuropsychiatric signs [99]. Neuropsychiatric signs refer first of all to anxiety, apathy and depression that are frequently encountered in de novo drug-naïve PD patients [54] and reverted when dopamine replacement therapy (DRT) is introduced [66, 81]. Neuropsychiatric signs also encompass neuropsychiatric fluctuations (NPF), Impulse control disorders (ICDs) and related disorders, and psychosis that are frequently observed along the progression of PD pathology and the concurrent pulsatile administration of increasing doses of DRT [25, 46, 52]. Therefore, behavioural disorders in PD have been conceptualized as a hypodopaminergic behavioural syndrome where apathy predominates, hyperdopaminergic behavioural syndrome which includes ICDs and other behavioural addictions, and non-motor fluctuations [2, 75] that together, define two opposite sides of one behavioural spectrum [81]. ICDs and related behaviors comprise a set of behaviors characterized by both impulsive and compulsive aspects. Impulsive aspects refer to an inability to resist an impulse or inappropriate drive despite harmful consequences for patients and their relatives, whereas compulsive aspects refer to the intrinsic repetitive nature of these behaviors that indicate a lack of self-control [100]. As with drug addictions, ICDs and related disorders are underpinned by one common pathophysiological mechanism that consists in hypersensitization of reward circuits and heightened ventral striatal dopamine release in response to reward-related cues [52]. Thus, from a clinical perspective, pathological gambling has been removed from ICDs in DSM-IV diagnostic criteria to be included into behavioural addictions in DSM-V. However, DSM-V nosology of ICDs and related disorders remains unclear and shared between ICDs and behavioural addictions. Based on the pathophysiology of those disorders, we prefer the label of behavioural addictions to describe the full spectrum of hyperdopaminergic behaviours [6, 52]. Dopamine dysregulation syndrome (DDS) is a specific entity characterized by compulsive use of levodopa irrespective of motor-fluctuations and dyskinesias, along with behavioural changes from the spectrum of behavioural addictions [30, 47]. Although DDS was initially thought to be driven by the severity of non-motor OFF symptoms such as pain or anxiety, a recent study argued that the non-motor “ON-drug” state was the driving force that enhance compulsive drug intake [20]. Thus, patients who developed DDS might be seeking “ON-drug” euphoria rather than preventing “OFF-drug” dysphoria [20]. Behavioural addictions result from complex interaction between individual risk factors, PD pathology and DRT. The purpose of the present review is to delineate the neurobiology and the clinical spectrum of behavioural addictions that are still under recognized despite their potential devastating consequences for patients and their relatives [69].

### Neurobiology

#### Brain systems involved

**Fig. 1:**
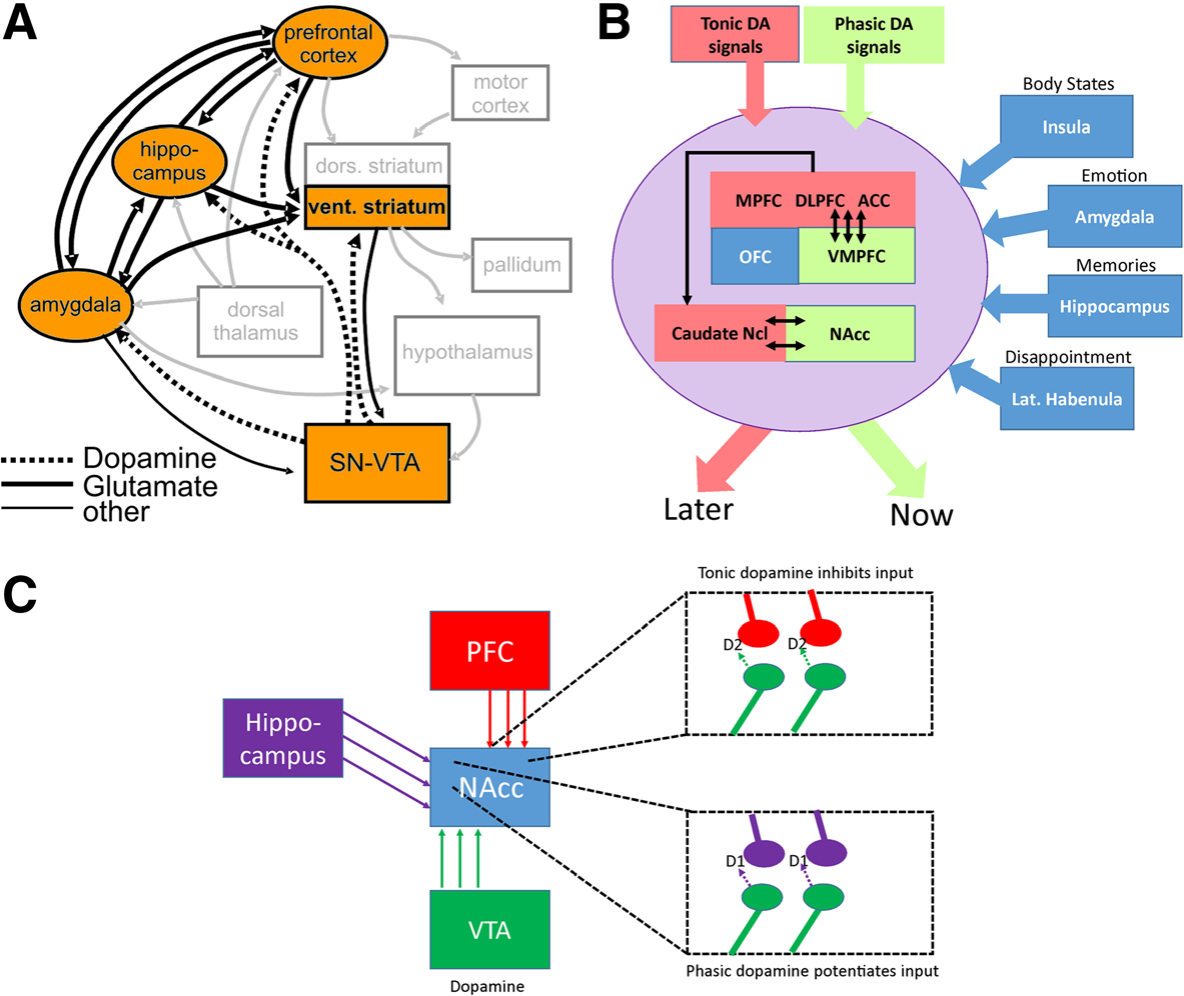
**a** Scheme of the reward valuation network as derived from a neuroimaging study by Camara et al. [11] (orange boxes, black arrows) embedded in a wider motivation/learning circuit (gray boxes and arrows). The wider network is based on Kelley et al. (2004), omitting unspecific hypothalamic/thalamic projections. **b** Model of intertemporal choice behavior as proposed by Volkow and Baler [93]. The regions colored in red are considered to support decisions for later, larger rewards (“LATER”), whereas the green areas support decisions for immediately available rewards (“NOW”). Regions depicted in blue modulate intertemporal choice behavior by integrating different information (described in black lettering). Please note, that tonic dopamine (DA) signals are thought to favor LATER rewards by influencing frontal regions. By contrast, phasic DA signals drive decisions towards choosing the immediately available reward (NOW). Regions: dorsolateral prefrontal cortex (dlPFC), medial PFC (mPFC), ventromedial PFC (vmPFC), orbitofrontal cortex (OFC), anterior cingulate cortex (ACC), nucleus accumbens (NAcc). **c** Illustration of the role of phasic and tonic dopaminergic projections (after [32]): The Nacc serves as an integrator of afferent inputs from frontal and limbic regions. Of note, the input from the PFC is regulated by dopaminergic input from VTA via presynaptic D2 receptors, with D2 receptor stimulation resulting in an inhibition of PFC input to the Nacc. The presynaptic neurons are stimulated by tonic dopamine neuron firing leading to low tonic levels of dopamine. High-amplitude, phasic dopamine signals on the other hand lead to D1 receptor activation that potentiates the hippocampal input to the NAcc

The mesolimbic dopaminergic system consisting of the dopaminergic ventral tegemental area (VTA) and the ventral striatum, with the nucleus accumbens as most prominent player, has been frequently dubbed as the brain’s reward system [38]. This view, however, falls considerably short of the complexity of the brain areas involved in guiding goal directed behavior [34, 36]. For example, Kelley [40] has compiled a complex network of brain areas including the mesolimbic dopaminergic system from animal work on ingestive behavior. Interestingly, we have been able to detect most of these brain areas in a neuroimaging study involving a gambling task ([11]; Fig. 1a). Inspection of this network suggest that the prefrontal cortex might act as a control instance. Indeed, ICDs in PD and in other conditions might be conceptualized as resulting from an imbalance between the craving for a reward and the inability to suppress this behavior, whenever it might not be appropriate.

#### Intertemporal choice task

To assess the interplay of the brain areas depicted in Fig. 1 and their involvement in ICDs in PD, the intertemporal choice paradigm has been used which involves choices between two rewards available at different times. Usually, this is an immediately available smaller reward and a larger reward which can be obtained only after a delay. A multitude of studies in humans and animals suggests that future are discounted in a quasi-hyperbolic or hyperbolic fashion [28, 57], the latter of which can be captured by the following function:

$$ V=\frac{A}{1+ kD} $$

where V is the present value of the delayed reward A after a delay D, and k is the *delay discount rate*. A greater delay discount rate indicates a steeper discount function, i.e. a more pronounced devaluation of future rewards. The neural underpinnings of intertemporal choice have been investigated using neuroimaging studies in humans and neurophysiological recordings in primates and other species [7, 23, 24, 35, 37, 55, 58, 59, 68, 109]. One of the earliest neuroimaging studies of intertemporal choice proposed two neural systems in intertemporal choice [58, 59]: a beta system, comprising limbic structures thought to place special weight on immediate rewards, whereas prefrontal cortical structures, the delta system, are thought to mediate deliberate, patient choices. Alternatively, a unitary system comprising medial prefrontal and posterior cingulate cortex and the ventral striatum has been proposed which represents and compares the values of both, immediate and delayed choices (Kable and Glimcher, 2007Peters and Büchel, 2009). Volkow and Baler [93] have recently pointed out that decisions for immediate or delayed rewards (in their words “now” or “later” processes) are differentially modulated by dopamine signals. Whereas decisions for delayed rewards require steady, tonic firing of dopaminergic neurons in striatal and prefrontal regions to sustain effort, “now” processes are thought to be dependent on fast, burst-like firing of dopaminergic neurons in ventral and dorsal striatal regions, which drives the desire to attain and consume the stimulus. This model is supported by brain imaging studies [27, 94].

Voon et al. [96] employed an intertemporal choice task in PD patients with ICDs, PD patients without clinically apparent ICDs and healthy controls. Patients were tested on and off dopamine replacement therapy. Of note, the task used by Voon et al. [96] employed rather delays (7–28 s) and real-time consummatory feedback during the task rather than long delays of weeks and months used in most behavioral economics tasks [41]. A group by medication interaction effect was revealed, reflecting a steeper discounting curve (i.e. more impulsive choice) in PD patients with ICDs when on dopamine agonist therapy. No medication effect was found in PD patients without ICDs. By contrast, Milenkova et al. [60] found a steeper discounting for PD patients in a monetary intertemporal choice task employing delays of up to 6 months. The PD patients in that study were not having clinically apparent ICDs. In a small study of 7 presymptomatic carriers of duplications of the SNCA gene who later developed PD, Szamosi et al. [85] found a similar delay discounting behavior during the presymptomatic stage in carriers compared to non-carrier control participants. After the development of PD a significantly steeper discounting (i.e., more impulsive decisions) were seen in the subjects with SNCA duplications after the initiation of dopamine replacement therapy. Again, these patients did not exhibit overt ICDs. It thus appears that impulsive choices are more abundant even in PD patients without ICDs.

#### Dopamine agonists lead to a blunted response of the reward system

**Fig. 2:**
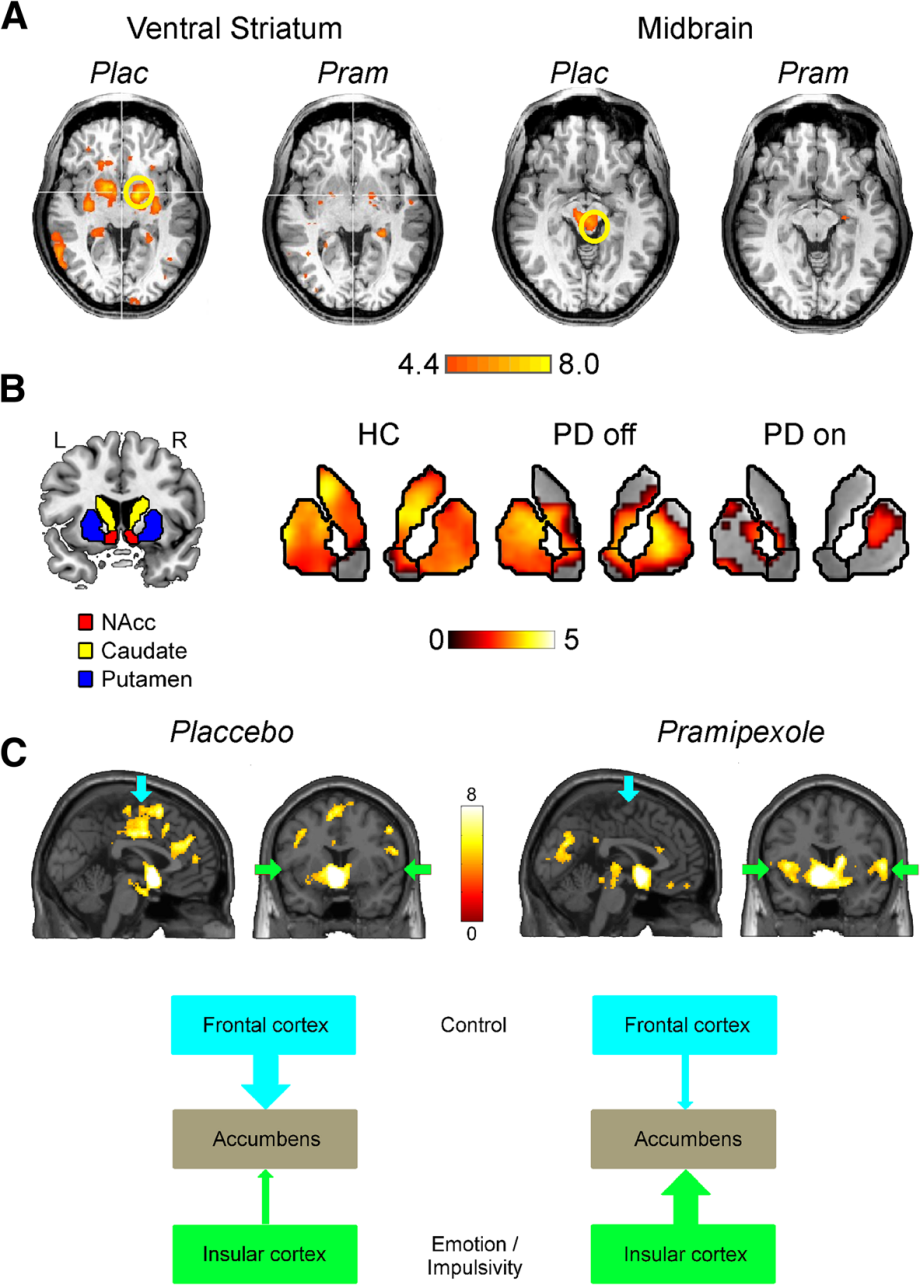
**a** FMRI results from a gambling task in young healthy participants. Axial slices show greater activation for win compared to loss trials in the ventral striatum and midbrain after placebo and pramipexole. A single dose of pramipexole (0.5 mg) resulted in a marked attenuation of reward based activations. After data presented in Riba et al. [74]. **b** FMRI results from a monetary incentive delay task. Shown are core regions of the reward processing network for the contrast “expectation of reward > expectation of no-reward”. Please note that PD patients on dopamine agonist treatment show a marked attenuation of activation compared to healthy controls and PD patients off medication (unpublished data from Ye and Münte obtained from 17 PD patients and 17 matched control participants). **c** Nucleus accumbens connectivity during reward expectation in a monetary incentive delay task. Regions functionally connected with the NAcc during reward expectation under placebo and pramipexole. Arrows indicate the frontal cortex (blue) and the insular cortex (green). The scheme at the bottom presents the connectivity patterns under placebo and pramipexole. The dopamine agonist therapy results in a shift of connectivity (less connectivity between NAcc and frontal cortex, greater connectivity between NAcc and insular cortex)

Clinical experience and large epidemiological studies (e.g., [102]) point to dopamine agonists as predisposing factors for the development of ICDs in PD. The question arises, how these drugs may modulate responses of the reward system. Importantly, Schott et al. [79] could demonstrate that reward-related ventral striatal dopamine release predicted functional magnetic resonance imaging activations in the ventral striatum during reward anticipation. Knutson and Gibbs [42] suggested that dopamine released in the Ncl. Accumbens (NAcc) changes the postsynaptic membrane potential by activating dopamine D1 receptors which in turn increases the local blood oxygen level dependent (BOLD) signal detected by fMRI. Thus, NAcc activity in response to possible rewards may be regulated by dopamine autoreceptors D2 and D3, which inhibit dopamine synthesis and/or release [22]. Dopamine D2/D3 receptor agonists then decrease NAcc dopamine release and reduce the incentive effect of rewards. Consistent with these predictions, we could demonstrate a blunted response to reward delivery in a gambling task in normal participants after a single dose of pramipexole ([74], Fig. 2a). Also, activation of the ventral striatum / NAcc was found reduced during anticipation of a reward in a monetary incentive delay task [106]. In Parkinson’s disease, fMRI activations to anticipated rewards were reduced in patients on dopaminergic agonist therapy but not patients off therapy (Fig. 2b). In addition to changing the response in the NAcc, dopaminergic agonist therapy also leads to profound changes of connectivity: Connections between the dorsolateral prefrontal cortex and the NAcc are downregulated after pramipexole, whereas connections between the insula and the NAcc are upregulated ([11]; Fig. 2c). Van Eimeren et al. [91] used H_2_^15^O PET before and after administration of 3 mg apomorphine to assess regional cerebral blood flow during a gambling task in PD patients with pathological gambling and PD patients without ICDs. Indeed, gamblers showed a significant DA-induced reduction of activity in reward related brain areas, corroborating the previously mentioned results. PD patients with ICDs have also been studied using radioligands binding to dopamine receptors. For example, Stark et al. [84] used [^18^F] fallypride, a high affinity D2-like receptor ligand in PD patients with and without ICDs. Patients with ICD had reduced binding potential in the ventral striatum and ICD symptoms positively correlated with midbrain D2/3 receptor binding potential. The authors suggested that ICDs in PD are associated with reduced ventral and dorsal striatal D2/3 expression, which may account for their differential response to dopamine agonist therapy.

#### Genetics

As not all PD patients on DRT develop behavioral addictions / ICD, the question arises whether a specific genetic background disposition might contribute to such behaviors. Multiple studies have been conducted to pinpoint single nucleotide polymorphisms (SNPs), mostly in genes related to the function of monaminergic transmitter systems, as either conducive or protecting. Evidence suggests a role of SNPs in genes involved in dopamine metabolism (COMT, DAT), dopamine receptors (DRD1, DRD2, DRD3, DRD4), serotonin receptors (HTR2A) and transporters (5HTT), and glutamate receptors (GRIN2B) [48, 49, 108], even though some studies also were negative [90]. These studies suggest that genetical factors play an important role in behavioral addictions in PD. However, as in other cognitive domains (e.g., executive functions, [45]) each SNP may account only for a small portion of the variance.

Therefore, a recent prospective multigene study by Kraemmer et al. [44] is of utmost importance. In this study, de novo drug-naïve patients (*n* = 276) with PD who had no behavioral addictions / ICD at the initial measurement were followed for 3 years. During this period 238 patients started DRT of whom 40% were taking a DA. During the follow-up, 19% of the patients developed behavioral addictions / ICD as determined by the Questionnaire for Impulsive-Compulsive Disorders in PD (QUIP). Heritability of such behaviors was estimated to be 57% by restricted maximum likelihood analysis on whole exome sequencing data. Importantly, the occurrence of behavioral addiction in a given patient could be predicted with 87% accuracy in patients receiving DA when a model contained clinical data and the genotypes of 13 candidate variants in the DRD2, DRD3, DAT1, COMT, DDC, GRIN2B, ADRA2C, SERT, TPH2, HTR2A, OPRK1 and OPRM1 genes. This compared to 71% accuracy based on clinical data alone. A backward stepwise regression analysis identified age, male sex, dopamine replacement therapy and ADRA2C, DRD2, DDC, HTR2A and OPRK1 genotypes as significant predictors of behavioral addiction / impulse control disorders. Taken together, this suggests that it might be possible in the future to predict which patients are vulnerable to behavioral addictions prior to initiation of DA therapy.

#### Beyond the dopaminergic system

A number of animal studies [4, 21] as well as work in human PD patients [31, 92, 98] have suggested a role for noradrenaline in impulsivity and inhibitory functions in addition to the undisputed function of the dopaminergic system. These findings have triggered studies using the selective noradrenaline reuptake inhibitor atomoxetine in PD to modulate the response inhibition system [10, 39, 73, 105, 107]. For example, Ye et al. [105] employed the stop-signal task in PD patients and control participants and found that the former had longer stop-signal reaction times as well as less stop-related activation in the right inferior frontal gyrus in fMRI accompanied by weaker functional connectivity between this region and the striatum. These changes in PD were normalized by atomoxetine. Rae et al. [73] using the same task proposed that atomoxetine acts by increasing sensitivity of the inferior frontal gyrus to afferent inputs from the pre-supplementary motor cortex. Warner et al. [98] reviewed the evidence for an effect of atomoxetine in clinical symptoms of impulsivity, risk taking, and global cognition. The few available studies indeed suggest a beneficial effect.

### Clinical features

#### Epidemiology

Prevalence of ICDs and related behaviors ranged from 13,6 to 33% according to studies [1, 5, 95, 97, 102]. This variability is partly related to declarative and cultural bias, clinical scales applied to asses ICDs, as well as methodological issue: cross sectional versus prospective studies, the clinical spectrum considered (i.e. the four ICDs strictly speaking or the whole hyperdopaminergic behavioral spectrum) and differences in populations investigated (de novo PD, surgical candidates or advanced PD). All together, these studies pointed out the close interaction between individual susceptibility, DRT and PD pathology in the emergence of behavioral addictions. Patients with ICBs and related behaviors were more likely to have anxious mood phenotype, more motor fluctuations, and to be younger [95, 97, 102]. Those results have been confirmed by the prospective ICARUS study where ICD positive patients were more likely to be male, younger at PD onset, have a longer disease duration, depressive symptoms and poorer quality of life [1].

As mentioned above, some recent genetic studies argued for a genetically determined risk factors for developing ICDs [17]. Polymorphisms in genes coding for dopamine metabolism, serotonine and glutamate receptors have been particularly studied [17]. Moreover, a large prospective study based on a candidate gene multivariable panel identified OPRK1 (involved in the opioid system) and DDC (dopamine system) polymorphisms as risk factors for ICDs and related disorders [44].

Dopamine agonist (DAs) exposure is the main risk factor of developing ICDs [100]. Since the DOMINION study, a drug class relationship has been postulated as DAs was associated with 2 to 3,5-fold increased odds of having an ICDs [102]. Those results were confirmed by further prospective studies [5, 18]. Recently, a longitudinal study conducted in 411 patients showed that lifetime average daily dose and duration of treatment were independently associated with ICDs with significant dose-effect relationships [18]. DA that exhibit a higher selectivity for D2/3 receptors mainly expressed within the mesocorticolimbic pathway, play a crucial role in the emergence of behavioral addictions [33, 81]. Furthermore, the pulsatility of DRT involved in sensitization of D1 receptors within the nigrostriatal pathway and dyskinesias [70], may be implicated as well in sensitization of D3 receptors within the mesocorticolimbic pathway and behavioral addictions [20, 77, 81]. Short acting, high potency DAs but also levodopa are associated with punding and DDS [26, 47].

A case-control study comparing de novo untreated PD patients and unmatched healthy controls investigated the question whether PD itself could confer an altered risk for ICDs and related behaviours [104]. No significant difference between the two groups has been shown regarding the prevalence of any ICDs valued at about 20%. The conclusion of this study was that PD alone, did not seem to provide an increased risk of developing ICDs [104]. However, once treatment is started, PD patients who have a more severe dopaminergic denervation as shown by DAT-scan are at higher risk to develop ICD. Thus, interaction between PD pathology and dopamine replacement therapy play a crucial role in the development of ICDs [82].

Behavioral addictions are more frequent in advanced PD and surgical DBS candidates [8, 25, 52]. Interestingly, advanced PD and surgical DBS candidates had higher disease duration, higher levodopa and agonist equivalent daily dose, and higher proportion of motor and non-motor fluctuations [8, 25, 52]. Moreover, multiple behavioral addictions have been frequently reported [25]. In the ALTHEA study, behavioral addictions were observed in 55% of PD patients with dyskinesias and motor complications [8]. Furthermore, frequency of ICDs symptoms was much higher in patients with severe dyskinesias compared to patients with mild to moderate dyskinesias. Dyskinesias occur in patients with high L-dopa sensitivity and result from a treatment, that is too pulsatile. Indeed, fractionating L-dopa or administering L-dopa with continuous perfusion can reverse dyskinesias and ICD [14]. Taken together, these observations support that pulsatility of DRT is another important common risk factor for both dyskinesia and ICD [8].

Neuropsychiatric fluctuations (NPF), that reflect mesocorticolimbic denervation along the progression of PD pathology, are associated with the emergence of behavioral addictions such as DDS in surgical DBS candidates [20], as well as post-operative withdrawal syndrome in post-operative DBS patients [86]. These results support a more complex interaction between DRT and PD pathology in the emergence of behavioral addictions. While non-motor fluctuations and ICDs are markedly reduced during the post-operative state in DBS PD patients [52, 53], DBS PD patients frequently develop post-operative apathy unmasked by the decrease in DRT, and related to desensitization of a more severe denervated dopaminergic and serotonergic mesocorticolimbic pathways [54, 86]. Thus, apathy and behavioral addictions may be considered as the Yin and Yang of dopamine dependent behaviors [81].

#### Clinical spectrum

The whole behavioral clinical spectrum illustrates the relation between personality, dopamine and behaviors [19, 51]. While PD patients frequently exhibit harm avoidance and introspective traits that refers to baseline personality traits [88], they also frequently develop apathy, anhedonia, depression, and anxiety during the premotor stage of the disease, the so-called “hypodopaminergic behavioural spectrum” [66, 80]. Conversely, later in the disease course, patients which are prone to develop ICDs and related disorders, the so-called “hyperdopaminergic behavioural spectrum”, frequently exhibit novelty seeking, and risk-taking personality traits, the exact opposite of the premorbid pattern mentioned above [19, 51].

The “honey moon” defines a well-being state for patients, where motor and non-motor signs are reverted by DRT [2]. The psychotropic effect of dopaminergic drugs might be the main factor that reflects this well-being feeling expressed by patients. During the motor and non-motor fluctuations state, neuropsychiatric fluctuations (NPF) are characterized by dysphoria, sadness, indifference, vulnerability in OFF state, and euphoria, pleasure, self-confidence in ON state [56]. NPF might play the driving force that promote behavioral addictions [20].

The hyperdopaminergic spectrum encompasses the four ICDs originally described, namely pathological gambling, hypersexuality, compulsive buying and compulsive eating as well as other behavioral addictions such as hobbyism, punding, walkabouts, hoarding, and DDS [100]. Pathological gambling predominates in men and is characterized by a preference for casinos and slots machine that can lead to financial problems. PD patients with hypersexuality had increased libido, increased desire for frequent sexual intercourse, as well as compulsive use of sex lines telephone, internet pornography, or prostitution services [30, 62]. Changes in sexual preferences and paraphilia have been more rarely reported [83]. Compulsive buying usually affects women and consists in an irrepressible and repetitive impulse to buy unnecessary items [16, 43]. Compulsive eating is defined by a persistent binge eating that occurs during day or night, with excessive and uncontrollable consumption of food [63]. Hobbyism that recently arose among the hyperdopaminergic behavioral spectrum is characterized by perpetual repetitive actions such as reading, internet browsing, working on projects, or painting [100]. Some other patients may also exacerbate or develop a creative art work [50]. Hoarding refers to a compulsive collecting of objects without objective value. In such cases, it can lead to unsanitary living conditions [64]. Punding is characterized by stereotypical motor behaviors such as manipulations, examinations, collecting, and other purposeless repetitive actions, that are frequently accompanied by dyskinesias and dopamine dysregulation syndrome (DDS), suggesting a common pathophysiological mechanism [26]. DDS is a clinical entity that correspond to an addiction to DRT combined with mood fluctuations and other behavioral addictions [30, 47]. Addiction to dopamine medications manifested by a compulsive craving and self-medication with increasingly dose of dopamine irrespective of motor state and dyskinesias [30, 47]. This DRT addiction, can lead to the full-blown DDS, where mood fluctuations and other behavioral addictions such as punding are present [30, 47].

#### Clinical tools

Different clinical tools have been validated for screening and/or diagnosis of ICDs and related behaviors. The Minnesota Impulsive Disorders Interview (MIDI) one of the first scale reported is not specifically addressed to PD patients [15, 16]. Other scales dedicated to PD have been developed: The Questionnaire for Impulsive Compulsive Disorders in Parkinson’s disease (QUIP) for screening [101] and the rating scale version of the QUIP, namely the QUIP-RS [103]. The Ardouin scale for behavioral assessment is based on a semi-structured interview that encompasses the whole spectrum of behavioral spectrum from hypo- to hyperdopaminergic syndromes as well as non-motor fluctuations [2, 75]. Non-motor fluctuations can be specifically addressed and quantified with the Neuropsychiatric Fluctuations Scale (NFS), a recently developed tool [78].

### Management of ICDs

Management of ICDs and related disorders remains challenging. Careful interview and education of patients and their relatives about risk factors: non-motor ON [20], dyskinesia [95, 97], DA intake (5 years cumulative risk of 50%) [18] is a crucial step before starting DRT [6]. Detection and evaluation of early changes in behaviors is mandatory when DRT is started. This consists of screening for benign changes along the behavioral spectrum from hypodopaminergia to hyperdopaminergia as well as neuropsychiatric fluctuations, using dedicated clinical scales [6].

When ICDs occur, practical management will be adapted, taking into account severity of ICDs and impact on QOL, rather than the semiology itself. Basically, DA dose reduction, fractionation of L-Dopa must be first considered. Importantly, clinicians have to bear in mind that desensitization of the mesolimbic dopaminergic system is prolonged and may take weeks or months [12, 13, 52, 86]. In such cases, adjunction of clozapine, amantadine or naltrexone may be helpful. Moreover, in cases of ICDs associated with DDS and severe motor fluctuations in younger PD patients, STN-DBS may represent an interesting therapeutic option.

DA should be carefully tapered below the individual threshold of inducing behavioral addictions, while avoiding the appearance of dopamine withdrawal syndrome [72]. Continuous delivery of D2/D3 agonists using extended release formulations or transdermal administration should be preferred [29], but overall all available dopamine agonists are very similar in effect. Apomorphine is the only available exception, as this agonist has a D1/D2 profile similar to endogenous dopamine with effects similar to L-dopa. Constant delivery of apomorphine with subcutaneous infusion using a mini-pump is a good alternative [3, 89]. Although typically not mentioned in the literature as evidence based studies are lacking, L-Dopa fractionation that is indicated when motor complications and dyskinesia occur, should also be considered for the management of non-motor complications and behavioral addictions as the first approach in patients on L-dopa. Although studies are lacking, based on expertise this is the easiest and most effective approach. Efficacy of non-pulsatile administration of L-dopa has been shown in a prospective observational study of 66 consecutive PD patients treated with levodopa-carbidopa intestinal gel infusion. Catalan et al. [14] found a significant 64,4% reduction of ICDs symptoms compared to baseline over the 6-month’s follow-up.

Clozapine, an atypical neuroleptic that improve levodopa induced psychosis and dyskinesia, has been reported to reduce ICDs symptoms in patients in which dopamine dose reduction did not improve those symptoms [9, 76]. Conflicting results have been shown with amantadine, a glutamate receptor antagonist. Although one small double blind crossover study reported improvement in pathological gambling in PD patients [87], the DOMINION study highlighted a positive correlation between amantadine and ICDs, even when taking into account levodopa and DA dosages [102]. One small intervention study with the opioid antagonist naltrexone showed some evidence for an effect [67]. This effect was corroborated by small clinical trials in other, non-PD-related behavioral addictions [61]. Accordingly, additional data are needed to support the use of clozapine, amantadine or naltrexone for the treatment of ICDs and related disorders. STN-DBS is a well-established treatment for PD patients with motor complications. Moreover, drastic dopamine dose reduction following STN-DBS is responsible for substantial decrease of dyskinesia. Recently, a randomized controlled study comparing STN DBS plus medical therapy and best medical treatment over a period of 2 years, demonstrated a better behavioral outcome with STN DBS plus medical therapy compared to best medical treatment [53]. Finally, the potential usefulness of cognitive behavioral therapy (CBT) for ICDs and related disorders has been highlighted in one randomized study [65].

## Discussion and outlook

Behavioral addictions / ICDs present a major problem in the clinical management of PD. Research over the past 20 years has revealed that occurrence of such behaviors is by no means an exceptional event [102] and that ICDs are but one aspect of a larger spectrum of hyperdopaminergic behavioral disorders that also include punding and dopamine dysregulation syndrome [6, 30, 47, 52]. Both dopamine agonists with a high affinity for the mesolimbic D3 receptor and pulsatile treatment with L-dopa contribute to ICD in interaction with the severity of the disease explaining that ICD can appear after many years of treatment and increase in DRT is not a mandatory prerequisite. Clinicians have to screen systematically for behavioral side effects of dopaminergic treatment using specific tools from initiation of therapy to the most advanced stages.

Neuroimaging approaches have only begun to unravel the neural underpinnings of these behaviors. A blunted response of the reward system comprising the ventral striatum as a core structure and a changed connectivity pattern are induced by dopamine agonists [11, 74, 106]. This shifts the balance towards more impulsive, risk seeking behaviors as evidenced by steeper discounting of future rewards seen most prominently in PD patients with ICDs [96]. A shift from the LATER system (in the sense of [93]) driven by tonic dopamine signals to the NOW system supported by phasic dopaminergic transmission underlies this preference for more impulsive behavior. These findings might be instrumental in the development of therapy strategies. Moreover, the finding of a substantial genetic contribution, i.e. predisposition, to the development of ICDs [44] might allow to taylor the dopamine replacement therapy according to the individual genetic profile.

## Abbreviations

DA: Dopamine agonists
DBS: Deep Brain Stimulation
DDS: Dopamine dysregulation syndrome
fMRI: functional magnetic resonance imaging
ICDs: Impulse control disorders
MIDI: Minnesota Impulsive Disorders Interview
NFS: Neuropsychiatric Fluctuations Scale
NPF: Neuropsychiatric fluctuations
PD: Parkinson’s disease
PET: Positron emission tomography
QUIP: Questionnaire for Impulsive Compulsive Disorders in Parkinson’s disease
QUIP-RS: Questionnaire for Impulsive Compulsive Disorders in Parkinson’s disease Rating Scale
